# *Mycobacterium tuberculosis* Resides in Macrophages in Laryngeal Tuberculosis: A Case Report

**DOI:** 10.3390/pathogens12121413

**Published:** 2023-11-30

**Authors:** Wafaa Achache, Mahmoud A. Boualam, Nadim Cassir, Clémence Mimari, Delphine Poitrenaud, Soraya Mezouar, Jean Louis Mège, Michel Drancourt, Hubert Lepidi

**Affiliations:** 1 MEPHI, Aix-Marseille University, IRD, 13005 Marseille, Francecassirnadim@gmail.com (N.C.); soraya.mezouar@univ-amu.fr (S.M.); jean-louis.mege@univ-amu.fr (J.L.M.); michel.drancourt@univ-amu.fr (M.D.); 2 IHU Méditerranée Infection, 13005 Marseille, France; 3 Service ORL Hôpital de la Conception, 13005 Marseille, France; 4 Service Maladies Infectieuses, CH Ajaccio, 20000 Ajaccio, France; delphine.poitrenaud@ch-ajaccio.fr

**Keywords:** larynx, tuberculosis, granuloma, *Mycobacterium tuberculosis*, laryngitis, extrapulmonary tuberculosis

## Abstract

Laryngeal tuberculosis is a rare form of extrapulmonary tuberculosis that questions the natural history of this infection. We report one such case in which a pathological examination of a laryngeal biopsy revealed granulomatous inflammation with caseous necrosis. Further investigations combining immunofluorescence detection of macrophages and in situ hybridization of *Mycobacterium tuberculosis* indicated the presence of *Mycobacterium tuberculosis* (*M. tuberculosis*) in laryngeal granulomatous inflammatory lesions. This observation suggests that the natural history of laryngeal tuberculosis does not differ from that of other forms, guiding early diagnosis in patients with laryngeal lesions to ensure appropriate check-ups and treatment.

## 1. Introduction

Tuberculosis is a lethal contagious infectious disease caused by members of the *Mycobacterium tuberculosis* complex, more specifically *M. tuberculosis stricto sensu* [1]. The lungs are the most common site for tuberculosis, reported in 80% of cases, but *M. tuberculosis* complex mycobacteria can reach extrapulmonary organs and tissues, including, among others, lymph nodes, bone, and meninges. Laryngeal localization is a rare form of tuberculosis, reported in approximately 1% of cases [2], in which laryngeal tuberculosis is most often associated with pulmonary tuberculosis [3,4,5].

Despite such an association, laryngeal examinations are not usually carried out in patients diagnosed with pulmonary tuberculosis, so the actual incidence of laryngeal tuberculosis is probably underestimated [6]. In fact, laryngeal tuberculosis is the most common granulomatous disease of the larynx [7]. It is highly contagious, and can present very Checkeddifferent aspects [8]. Symptoms appear gradually, ranging from hoarseness to difficulty swallowing, and the diagnosis is usually delayed [9]. Its rare appearance explains why it is often overlooked when vocal difficulties are diagnosed [10], challenging the differential diagnosis consisting of non-specific laryngitis, granulomatous diseases and tumors of the larynx [11]. Appropriate management involves tissue biopsy followed by pathological examination for the presence of caseous granulomas and detection of the causative agent, *M. tuberculosis* [12].

We report a rare case of laryngeal tuberculosis in which cellular localization of *M. tuberculosis* was demonstrated in tissue lesions. 

## 2. Case Presentation

A 68-year-old non-smoker born in Algeria and living in Corsica, France, for 30 years was seen for dysphonia. The patient, who gave informed written consent for anonymously reporting the case, had no significant medical history. He had dysphonia for five months, worsening for two months, with progressive dysphagia and odynophagia associated with productive cough. During this period, the patient did not report weight loss, fever, sweating, or chills and denied any recent travel or known exposure to tuberculosis. At the time of admission, the patient was apyretic and physical examination was notable for multiple non-tender mobile lymph nodes of the right posterior cervical chain; no pharyngeal exudate or erythema was noted. White blood cell (WBC) count was 13,500 μL, platelet count was 261,000 μL, and HIV serology was negative. A computed tomography (CT) scan identified mediastinal and hilar adenopathy, including necrotic adenomegaly at the left jugulo-carotid level of sector IIa with infiltration of adjacent fat and bilateral, predominantly right mediastinal–hilar adenomegaly, as well as solid nodules in both lung apices. Microbiological investigations yielded Ziehl–Neelsen-stained mycobacteria identified as *M. tuberculosis* complex by real-time polymerase chain-reaction (GeneXpert, Sunnyvale, CA, USA) in three respiratory tract specimens along with a laryngeal biopsy made by tracheotomy, as previously described [13]. Furthermore, six respiratory tract specimens and one stool specimen cultured colonies identified as *M. tuberculosis* by using matrix-assisted laser desorption/ionization time-of-flight, as previously described [14]. The patient being therefore diagnosed with pulmonary and lymph node tuberculosis, was started on RIPE therapy (Rifampicin, Isoniazid, Pyrazinamide, and Ethambutol) for a period of two months, followed by seven months of therapy including Isoniazid and Rifampicin. The one-year follow up indicated relief of complaints and resolving cough and lymph nodes with complete healing. 

**Figure 1:**
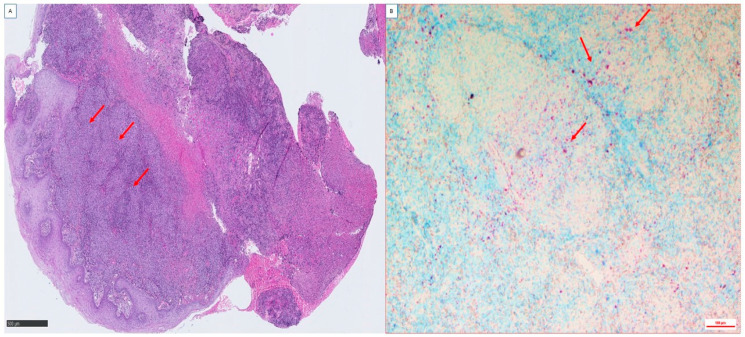
Microscopic observation of a hematoxylin–phloxine–saffron-stained laryngeal biopsy from a patient with laryngeal tuberculosis. (**A**): Observation of very inflamed tissue with areas of necrosis, and the presence of tuberculoid granulomas composed mainly of macrophages, lymphocytes and giant cells (red arrow). (**B**): Ziehl–Neelsen staining demonstrates the presence of *M. tuberculosis* (red arrow). Scale bars indicate 500 µm. Original images were made by the authors.

**Figure 2:**
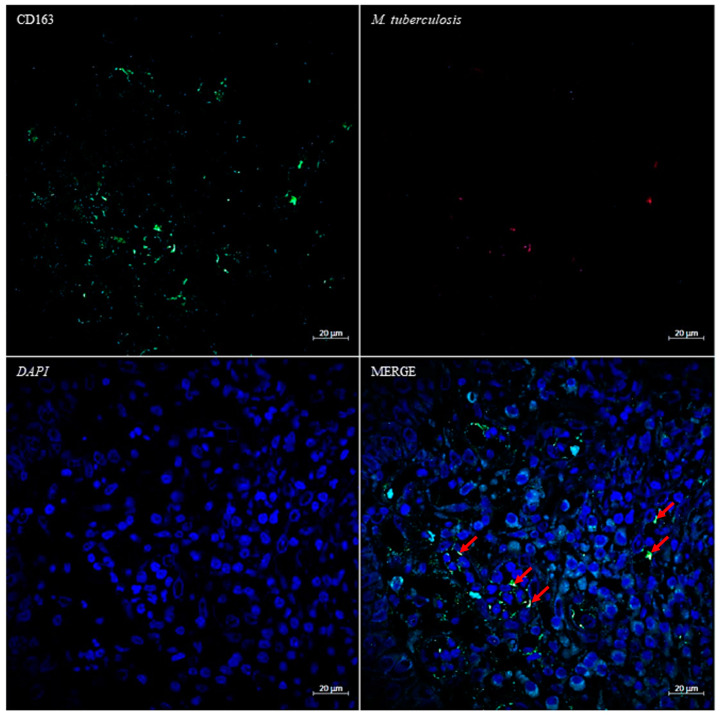
Fluorescence microscopic images of patient biopsy sections after combined immunofluorescence–fluorescence in situ-hybridization techniques: in green, antibodies labeled Alexa 488 anti-CD-163 for macrophage staining. In red, the specific probe of *M. tuberculosis* after FISH. In blue, DAPI for nucleus staining. The colocation of the red and green signals is indicated by the red arrows. Original images made by the authors. Scale bars indicate 20 µm.

Formalin-fixed and paraffin-embedded laryngeal biopsy samples were cut to 3-µm thickness and stained with hematoxylin–phloxine–saffron. For each tissue sample, serial sections were also obtained to perform special stains and immunohistological investigations. Special stains that were used for the detection of bacteria and fungi included Giemsa, PAS, Grocott–Gomori methenamine silver, Warthin–Starry, and Ziehl–Neelsen acid-fast stains. Histological examination revealed epithelioid granulomas with giant cells and rare areas of caseous necrosis with polymorphic inflammation. The Ziehl–Neelsen staining revealed the presence of acid-fast bacilli disseminated throughout the laryngeal biopsy (Figure 1). To further investigate tuberculous granulomas, we carried out combined immuno-fluorescence using a specific anti-macrophage antibody and in situ fluorescence hybridization (FISH) using a specific *M. tuberculosis rpoB* DNA probe [15]. In brief, slides were incubated for 15 min with permeabilization buffer consisting of 10% fetal bovine serum and 0.1% Triton X-100 in phosphate-buffered saline (PBS, Thermo Fisher Scientific, Illkirch, France), then were washed three times with PBS, incubated with blocking buffer (1× PBS, 10% FBS) for 30 min and rinsed with PBS. Slides were then incubated with an anti-CD163 antibody (Jackson Immuno Research Laboratories, West Grove, PA, USA) diluted at 1:250 in 5% fetal bovine serum-PBS for one hour and washed three times with PBS. FISH was then performed as previously described [15]. After observation and acquisition of fluorescence signal by laser scanning microscopy, a red signal corresponding to *M. tuberculosis*-specific probe (561 nm laser) was observed surrounded by a green fluorescence (488 nm laser) corresponding to a macrophage CD163 signal with a blue (405 nm laser) mark showing large macrophage nuclei (Figure 2).

## 3. Discussion

While a few previously reported cases have been documented by pathological examination of Ziehl–Neelsen-stained laryngeal tissues allowed for microscopic detection of acid-fast bacilli in tissues [16]; in this patient, *M. tuberculosis* localized in macrophages constituting granulomas in tuberculous laryngitis. Conventional microscopic examination of the laryngeal biopsy specimen by Ziehl–Neelsen staining showed acid-fast bacilli scattered throughout the specimen, and advanced diagnostic combining FISH labeling of *M. tuberculosis* provided more specific detection and immunolabelling of macrophages: indeed, FISH, incorporating probes demonstrated to be specific for *M. tuberculosis*, ensures specificity of the identification (*M. tuberculosis*, in this case) over the Ziehl–Neelsen in staining all the microbes containing mycolic acid in the cell wall, and beyond [15,17]. This observation was carried out using appropriate techniques, including immunofluorescence–fluorescence in situ-hybridization, which allowed the findings to be validated.

This unique observation indicates that the pathology of laryngeal tuberculosis does not differ from that of pulmonary tuberculosis, two related forms of tuberculosis, suggesting a common natural history, relying on the colonization of tissues by the lymphatic pathway [18]. The portal of entry may indeed be the larynx itself [10], as laryngeal tuberculosis clinically appears as primary in 40% of laryngeal tuberculosis cases and is the unique involvement of the larynx in the absence of pulmonary tuberculosis. Alternatively, secondary laryngeal tuberculosis may develop by *M. tuberculosis* spreading from pulmonary tuberculosis [19].

At the end of the 19th century, laryngeal tuberculosis was the most commonly diagnosed laryngeal disease [20]. The advent of efficient anti-tuberculous therapies allowed for the early control of tuberculosis, particularly in its pulmonary form, and a considerable decrease in the frequency of laryngeal tuberculosis was observed [21]. Today, laryngeal tuberculosis is a rare form of extrapulmonary tuberculosis, observed in areas where tuberculosis is highly endemic [22]. The diagnosis of laryngeal tuberculosis is usually delayed due to unremarkable symptoms, while only severe symptoms lead patients to diagnosis following severe odynophagia and dyspnea caused by laryngeal edema and granulations [20]. The vocal cords remain the most frequent location, followed by the arytenoid cartilage and epiglottis [8]. The pathological hallmark of tuberculosis is the formation of caseous granulomas that may result in an infiltrating mass [23,24], with extremely rare cases being located only in the laryngeal organ. A high degree of suspicion is therefore necessary, especially in patients with risk factors for tuberculosis. The gold standard diagnosis relies on the demonstration of *M. tuberculosis* in a laryngeal biopsy. Further diagnostic tests such as culture and imaging studies can be useful in supporting the diagnosis [25]. The diagnosis of laryngeal tuberculosis is usually made based on a biopsy. In some cases, histopathological examination reveals epithelial giganto-cellular inflammation with caseous necrosis. Indeed, the discovery of lymphocytic laryngitis in a clinical context suggestive of tuberculosis should lead to a Ziehl–Nielsen stain and/or a culture of the biopsy fragments [26]. In a further series, the histological lesions were epithelial giganto-cellular granuloma in 100% of cases and caseous necrosis in 20% of cases [11].

## 4. Conclusions

In this patient, the diagnosis of a rare form of laryngeal tuberculosis through histological and microbiological examination showed morphological features similar to those observed in the most commonly infected organs and tissues such as lymph nodes and lung. This observation suggests that the natural history of laryngeal tuberculosis does not differ from that of other forms, being primarily an infection of lymphatic tissues and organs with diffusion to other tissues and organs. 

## Data Availability

All data deriving from this study are given in the article.
